# Coronary Stent Abscess in the Setting of Arteriovenous Graft Infection following COVID-19: An Autopsy Case Report

**DOI:** 10.1155/2023/9998749

**Published:** 2023-05-03

**Authors:** Jean Thompson Butler, Rajeshwari Chellappan, Silvio Litovsky, Sixto M. Leal, Paul V. Benson

**Affiliations:** ^1^University of Alabama at Birmingham School of Medicine, USA; ^2^University of Alabama at Birmingham Department of Pathology, USA

## Abstract

While rare, coronary stent infections present with significant mortality—with most infections and further complications occurring within months of percutaneous coronary intervention (PCI). Here, we discuss a post-COVID-19 patient who presented approximately one year after PCI for declotting of an arteriovenous graft (AVG). Upon admission, the patient was found to be bacteremic with multilobar pneumonia and an infection of the AVG. Empiric antibiotics were started, and blood cultures were subsequently positive for MRSA. Removal of the AVG was unsuccessful, and two days after admission, the patient passed. Autopsy revealed a perivascular abscess in the RCA near the origin of the stent with a ground section of the RCA with stent revealing abundant calcific atherosclerosis and marked necrosis of the artery wall. The cause of death was determined to be sepsis complicating coronary artery disease and chronic renal failure.

## 1. Introduction

Percutaneous coronary intervention (PCI) with stent placement is a well-developed and frequently used modality for treatment of coronary artery stenosis. Although coronary stent infection is a rare complication, it presents with significant morbidity and mortality [1]. We present a case of a post-COVID patient hospitalized for arteriovenous graft (AVG) infection and sepsis, who was subsequently found to have a coronary stent abscess upon autopsy approximately one year post PCI.

## 2. Case Presentation

A 48-year-old male with hypertension, diabetes, sarcoidosis, obstructive sleep apnea, obesity, chronic anemia requiring transfusions, chronic kidney disease, and history of renal oncocytoma status postpartial nephrectomy underwent a routine regadenoson stress test as part of preop renal transplant evaluation due to hypertensive cardiovascular disease and diabetes. The diabetes was well controlled for over a year by diet alone with a recent hemoglobin A1C of 5.5. Sarcoidosis was diagnosed and treated 4 years prior to death after being admitted for hypercalcemia with mediastinal, mesenteric, and retroperitoneal lymphadenopathy and a lymph node biopsy consistent with sarcoidosis. He underwent high-dose steroid therapy and methotrexate therapy with no further biopsies or treatment. That regadenoson stress test showed myocardial perfusion images consistent with a medium-to-large area of ischemia scar in the distribution of the left circumflex coronary artery (LCA), involving 17% of the left ventricular (LV) myocardium. A left heart catheterization (LHC) revealed severe stenosis of the proximal and midright coronary artery (RCA). PCI followed by stenting of the proximal to mid-RCA with two overlapping Synergy drug-eluting stents was performed. Two months post PCI, the patient presented to the emergency department with substernal, nonradiating chest pain that resolved with sublingual nitroglycerin and aspirin. No changes were noted on the ECG, but the patient was found to be hyperkalemic with metabolic acidosis. A right internal jugular PermCath was placed, and intermittent hemodialysis (iHD) was initiated.

Four months later—six months post PCI—the patient was admitted to the hospital due to ARDS secondary to COVID-19 (SARS-Cov-2) pneumonia and bacteremia. After three weeks, the patient was discharged to an inpatient rehab facility before returning home at baseline.

Nine months post PCI, the patient underwent a left AVG placement and venogram, with the previously inserted PermCath removed approximately one month later. Three months following left AVG placement—and one year post PCI—the patient presented for declotting of the AVG and iHD. A negative COVID test was obtained, and declotting began.

**Figure 1:**
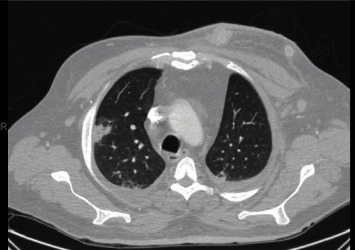
Scattered bilateral consolidations with an area of cavitation in the right upper lobe and left pectoral AV graft with skin thickening.

After declotting, difficulty maintaining access for iHD persisted, and iHD was stopped. Upon assessment, the patient was found to be febrile, tachycardic, and tachypneic, with decreasing blood pressure, a new oxygen requirement, and elevated troponin. The patient complained of shortness of breath, a nonproductive cough, diaphoresis, as well as severe left-sided flank pain that improved with pain medication. The ECG showed no change from baseline. A diagnosis of an NSTEMI type II in the setting of volume overload due to a lack of iHD was suspected. The chest CT obtained was remarkable for multilobar pneumonia with solitary focal area of cavitation in the right upper lobe (Figure 1).

Skin thickening and a small amount of fluid and subcutaneous gas were noted around the left chest wall AVG. Vancomycin and ceftazidime were started, and a central venous line was placed in the right femoral vein for iHD. Upon successful iHD, patient reported improvement in shortness of breath and oxygen requirement was decreased. Blood cultures were subsequently positive for MRSA bacteremia, concerning for an AVG infection.

The next day, complete graft removal was attempted unsuccessfully due to significant scarring and infection of the arterial and venous anastomoses. A left chest washout with antibiotic bead placement and temporary closure was completed instead. Swabs of the left chest graft were positive for MRSA. The patient returned to the OR the next day to undergo a second left chest washout with antibiotic bead replacement and temporary skin closure pending return to the OR for complete AVG removal. Due to consistent bacteremia, the patient was switched to daptomycin and ceftaroline in attempt to sterilize the blood stream. The patient reported chest pain that afternoon, but the ECG demonstrated minimal changes and sequential troponin levels were decreased in comparison to assessment at admission. The chest pain resolved following a decrease in volume via continuous renal replacement therapy. The next morning, the patient was found unresponsive, bradycardic, and hypotensive. Pulse was absent, and life-saving measures were initiated to no avail.

**Figure 2:**
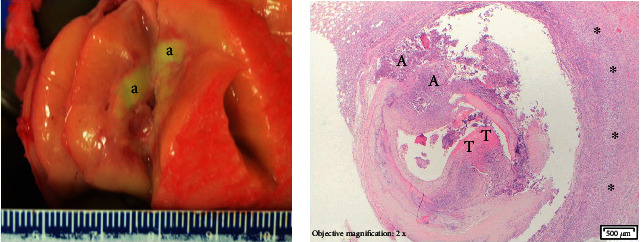
(a) Perivascular abscess (a) of right coronary artery adjacent to stent. (b) 2×, H&E right coronary artery immediately distal to stent with abscess, thrombus and wall destruction without aneurysm (T: thrombus; A: abscess; ^∗^transmural inflammation).

**Figure 3:**
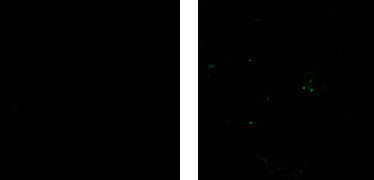
RNAscope for SARS-CoV-2 spike protein was negative in the RCA abscess (a). Positive control is shown (b).

Written consent for autopsy was obtained from the next of kin including consent for diagnostic, research, and educational uses; however, no written consent has been obtained from the patient, as there is no identifiable patient data included in this case report. Institutional review board approval was not required for this single patient case report. The decedent weighed 91 kg and was 180 cm in length. The body mass index at autopsy was 28.2 kg/m^2^. Three sutured incisions in the left pectoral area were over a 5 cm diameter arteriovenous graft pocket containing seven antibiotic-infused beads, granulation tissue, and no evidence of abscess. Histology of the left pectoral graft pocket showed increased acute inflammation and granulation tissue with hemosiderin-laden macrophages and polarizable foreign material. Autopsy showed bilateral pneumonia with pulmonary abscess formation. The lungs weighed 1280 g combined. There was 400 mL of left serous pleural effusion and 200 mL of right serous pleural effusion. There was no ascites. The heart weighed 620 g and had diffuse fibrinous pericarditis with 50 mL of serous pericardial effusion. A perivascular abscess was present involving the RCA, near the origin of the stent and showed thrombosis with focal vessel wall destruction (Figures 2(a) and 2(b)). The same section was submitted for RNAscope FISH analysis for SARS-CoV-2 spike protein (Bio-Techne, Cat# 848561). SARS-CoV-2 spike protein RNA was not detected by this method in the RCA abscess (Figure 3).

**Figure 4:**
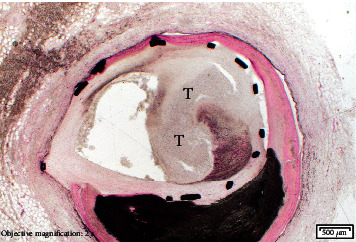
Transmural acute inflammation and intraluminal organizing thrombus (T) in the stented RCA. H&E 2× ground section.

A ground section of the RCA with a stent showed abundant calcific atherosclerosis and marked abundant necrosis of the artery wall with abundant transmural neutrophils leading to an intraluminal, partially occlusive thrombus (Figure 4).

**Figure 5:**
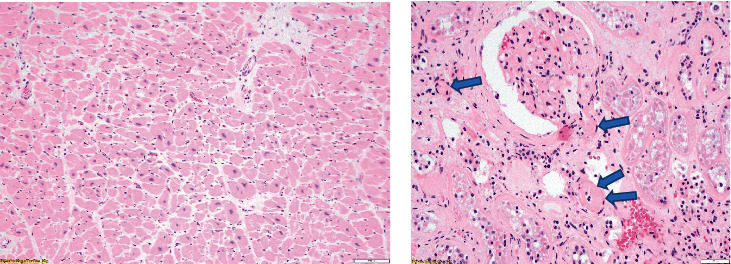
Myocyte hypertrophy in the heart (a) and hyaline arteriolosclerosis (arrows) (b) 10× H&E.

The left kidney weighed 180 g, and the right kidney weighed 200 g. Both are with mildly granular surfaces. Histologically, significant hypertensive and atherosclerotic cardiovascular disease were present in the heart and kidneys consisting of cardiomyocyte hypertrophy without acute hypoxic changes and renal arteriolosclerosis with a hyaline change (Figure 5).

Neuropathologic examination of the 1310 g brain showed mild cortical atrophy with mild to moderate hypertensive cerebral vascular disease and cavum septum pellucidum. The liver, gallbladder, spleen, intestines, stomach, and prostate gland were grossly unremarkable. Postmortem blood cultures were positive for *Bacillus species*, *Staphylococcus aureus*, *Pseudomonas aeruginosa*, *Staphylococcus capitis*, and *Staphylococcus epidermidis*. Although postmortem blood cultures showed multiple organisms, antemortem blood cultures and antemortem left pectoral AV-graft pocket cultures (both performed 4 days prior to death) showed only *Staphylococcus aureus*, consistent with *Staphylococcus aureus* sepsis. Given the clinical history and autopsy findings, the cause of death was determined to be sepsis complicating coronary artery disease and chronic renal failure.

## 3. Discussion

Although an increasing number of PCIs are performed each year, coronary artery stent infections continue to be a rare complication [1]. With a mortality rate greater than 40%, quick diagnosis and appropriate management are necessary [2]. Here, we present a patient with an infected AVG who was subsequently found to have a previously undiagnosed coronary artery stent abscess upon autopsy.

Of the coronary artery infections reported in the literature, most occurred within 4 weeks of stent placement and present with chest pain, fever, elevated troponins, and ECG changes [1, 3, 4]. Risk factors for infection have been noted to include older age, difficult vascular access, extended duration of the procedure, and repeated catheterizations using the same vascular access site [3, 5]. Although the pathophysiology is unknown, it is believed that stent infections—especially early stent infections—occur due to contamination or a distant source of infection at the time stent of insertion [1, 3]. Furthermore, it has been suggested that stents allow for bacterial colonization and that endothelialization of stents likely play an important role in the prevention of infection. This theory could explain why stent infections are observed more frequently in drug-eluting stents, as their antiproliferative effects delay endothelialization [1, 6].

This case is unique. In that stent abscess was identified at autopsy a year after uncomplicated PCI. Of note, our patient presented afebrile with chest pain 3 months after PCI, but no changes were seen on the ECG. The pain was relieved with nitroglycerin and aspirin, and the patient was found to be hyperkalemic with metabolic acidosis. When the patient did present with fever, it was only after infection of the AVG and bacteremia were present. During that same stay, patient's troponin was noted to be elevated, but again, ECG was unchanged from baseline, and NSTEMI in the setting of fluid overload due to a lack of iHD was suspected. With the underlying AVG abscess and bacteremia, the diagnosis of a stent infection was not made until autopsy. The patient had been treated for COVID-19 pneumonia and discharged from the hospital 166 days prior to his death. In order to help determine any contribution of COVID-19 to the death and contribute knowledge to the natural history of COVID-19 and potentially long COVID, postmortem testing for SARs-CoV-2 was performed. Postmortem testing for SARS-CoV-2 spike protein was negative in the coronary artery abscess; therefore, the abscess was unrelated to his previous COVID-19 infection.

## Data Availability

All data is contained in the manuscript.
